# Catheterization of Intestinal Loops in Ruminants

**DOI:** 10.3791/1301

**Published:** 2009-06-11

**Authors:** Richard R. E. Uwiera, John P. Kastelic, G. Douglas Inglis

**Affiliations:** Department of Agricultural, Food and Nutritional Science, University of Alberta, Edmonton; Agriculture and Agri-Food Canada, Lethbridge Research Centre, Lethbridge

## Abstract

The intestine is a complex structure that is involved not only in absorption of nutrients, but also acts as a barrier between the individual and the outside world. As such, the intestine plays a pivotal role in immunosurveillance and protection from enteric pathogens. Investigating intestinal physiology and immunology commonly employs 'intestinal loops' as an experimental model. The majority of these loop models are non-recovery surgical procedures that study short-term (<24 hr) changes in the intestine (1-3). We previously created a recovery intestinal loop model to specifically measure long-term (<6 mo) immunological changes in the intestine of sheep following exposure to vaccines, adjuvants, and viruses (4). This procedure localized treatments to a specific 'loop', allowing us to sample this area of the intestine. A significant drawback of this method is the single window of opportunity to administer treatments (i.e. at the time of surgery). Furthermore, samples of both the intestinal mucosa and luminal contents can only be taken at the termination of the project. Other salient limitations of the above model are that the surgical manipulation and requisite post-operative measures (e.g. administration of antibiotics and analgesics) can directly affect the treatment itself and/or alter immune function, thereby confounding results. Therefore, we modified our intestinal loop model by inserting long-term catheters into the loops. Sheep recover fully from the procedure, and are unaffected by the exteriorized catheters. Notably, the establishment of catheters in loops allows us to introduce multiple treatments over an extended interval, following recovery from surgery and clearance of drugs administered during surgery and the post-operative period.

**Figure:**
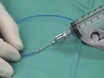


## Protocol

### Experimental procedure:

All instruments are autoclaved; non-autoclaveable materials are sterilized in Germex cold sterilization solution for a minimum of 10 min.
Sheep are pre-medicated with acepromazine (0.05 mg/kg), glycopyrrolate (0.005 mg/kg), and butorphanol tartrate (0.2 mg/kg) IM, and left for 15-20 min.
The neck is shaved and the skin surgically scrubbed. Once the jugular vein is visualized, 0.1 ml of lidocaine is injected SC over the vein, the catheter is introduced into the vein and secured to the skin using livestock identification tag cement and 2" hospital tape. During surgery, the animals are given 10 ml/kg/hr of Plasmalyte 148 (by gravity flow).
Diazepam (0.2 mg/kg) is administered IV, and anesthesia is induced with thiopental sodium (10 mg/kg). Lidocaine endotracheal spray is applied to the vocal folds, the sheep is intubated, and maintained on isoflurane (1.5-2.5% in 100% O_2_ at a rate of 4 L/min) for the duration of the surgery.
The animal is placed in dorsal recumbency on a heated mat to help maintain body temperature during surgery. A stomach tube is inserted into the rumen to prevent excessive tympany.
The ventral abdomen is shaved and the skin is prepared for surgery with three Prepodyne surgical scrubs. The abdomen is then draped, ensuring that appropriate exposure of the surgical site (umbilicus to caudal abdomen) is maintained.
A 15-20 cm midline incision is made using an electroscapel, caudally from the umbilicus, and the ileocecal fold is located in the right lower quadrant of the abdomen. The caudal ileum and cecum are then exteriorized. Throughout the procedure, handling of the intestine is minimized and care is taken to ensure the exteriorized intestine is kept moist with warm (37-39°C) sterile phosphate buffered saline (PBS; pH 7.2).
An 85 cm segment of ileum (i.e. the 'intestinal segment') is measured and then clamped with Doyen forceps (intestinal clamps) and large Kelly forceps at each end of the segment. Within the intestinal segment, 'loops' and 'interspaces' between the loops will be established. Doyen forceps are placed on the side of the ileum to be joined (anastomosis), and the Kelly forceps are placed on the intestinal segment side of the ileum.
The ileum is cut between each Doyen and Kelly forcep and the intestinal segment designated for loops is gently flushed twice with 60 ml of warm PBS to remove ingesta.
A cocktail of broad-spectrum antibiotics (200 mg enrofloxacin and 250 mg metronidazole in warm PBS, 60 ml total volume) is distributed throughout the intestinal segment and left for 30 min.
The proximal and distal cut ends of the ileum (excluding the intestinal segment) are joined to form a continuous and functional intestinal tract. First, the ileum is aligned by two stay sutures, placed at the mesenteric and antimesenteric borders of the intestine, respectively. Then, a simple continuous suture pattern (2-0 Vicryl) is completed on both sides of the intestine to join the intestine together.
Warm PBS (3-5 ml) is injected at the anastomosis site to check for leakage and suture failure. Then, the tip of Kelly forcep is pressed against the serosal surface of the intestine, close to the site of the anastomosis site, and advanced inwards at the anastomosis site to ensure the intestinal lumen is large and patent.
The antibiotic cocktail is removed from the intestinal segment. Each end of the segment is closed with a simple continuous suture pattern (absorbable suture; 2-0 Vicryl) followed by an inverting suture pattern using 2-0 Vicryl.
The 85 cm-long intestinal segment is partitioned into three 15 cm 'loops', two 15 cm-long 'interspaces' between the loops, and two 5 cm-long blunt-end compartments at the termini of the intestinal segment (Figure 1). The loops are created by placing a ligature around the intestine using 2-0 silk. It is noteworthy that major mesenteric vessels are not ligated during this procedure, as these are needed to supply the intestine with blood, maintaining intestinal viability. Also, the ligatures are left long, as they will be used to secure the catheters.
Catheters (i.e. silastic tubing) are individually identified using a permanent marker. A silicon ball (5 mm-diameter) encircling the catheter, is placed 4-5 cm from the catheter end to help prevent the catheter from sliding out of the intestine (Figure 1). After sterilization by autoclaving, a non-antibiotic ointment (Orbeseal) is aseptically inserted into the catheter end (3-5 mm); this ensures that the catheter remains patent (i.e. it does not become plugged by sloughed mucosa within the loop).
A small incision is placed at the cranial aspect of each of the three loops. Each hole is enlarged with a catheter introducer (i.e. 'vessel dilator'), and a 6-8 cm segment of catheter is inserted caudally into the loop, until the silcone ball passes into the lumen (Figure 1).
The intestinal wall is secured around the catheter with a purse string suture (2-0 Vicryl). The catheter is further secured to the intestine by tying the catheter with the ligature silk that delineated the cranial end of the loop.
Ampicillin (5 ml; 100 mg/ml in warm PBS) is injected into each loop and interspace, and the intestine is placed back in the abdominal cavity. Cefazolin (8 ml; 100 mg/ml in warm PBS) is injected into the peritoneal cavity through the abdominal incision.
A single small stab incision is made through the abdominal wall adjacent to the midline incision, and the catheters are exteriorized through this site.
The position and tension of the catheters is adjusted within the peritoneal cavity. Abdominal muscles are closed with a simple interrupted suture pattern (absorbable suture; Catgut #3), and skin is closed with an interrupted horizontal mattress pattern (non-absorbable suture;  Supramid #1).
A curved hollow stainless steel tube (7 mm ID; 10 mm OD; 65 cm in length) is inserted under the skin near the abdominal incision, tunneled under the skin to an exit site just caudal to the neck and between the shoulders. The catheters are inserted into the tube and pushed forward for at least 50% of the tube length. The tube with the catheters is then pulled from the skin at the exit site, exposing the catheters.
The tension of the catheters is adjusted at the stab incision site, and the skin is closed with Supramid #1.
Individual catheters are identified and placed within a bandage "pouch" which is sutured to the skin with Supramid #1. The pouch protects the catheters from subsequent damage.
Post-operatively, sheep are injected IM with flunixin (s.i.d.) at an initial dose 2.2 mg/kg, followed by a maintenance dose of 1.1 mg/kg s.i.d. for 3 days. Sheep also are injected IM with Hemostam (3 ml) and enrofloxacin (2.5 mg/kg) s.i.d. for 3 and 5 days, respectively. Animals are maintained on intravenous Plasmalyte 148 with 5% dextrose solution, administered continuously with a peristaltic pump to ensure that flow (145 ml/hr) continues regardless of body position. They are allowed to drink *ad libitum*, but feed is restricted until normal rumen and bowel functions are restored. Food intake, water consumption, body temperature, passage of feces and urine, gut noises, abdominal discomfort, demeanor, and blood glucose concentrations are closely monitored (b.i.d.); when necessary, glucose concentrations in the intravenous fluids are adjusted to maintain blood glucose concentrations within the normal range. Blood chemistry and hematology (i.e. albumin, alkaline phosphatase, alanine aminotransferase, amylase, calcium, cholesterol, creatinine, glucose, inorganic phosphate, total bilirubin, total protein, urea, globulin, sodium, potassium, chloride, partial pressure oxygen, pH, partial pressure carbon dioxide, bicarbonate, and total carbon dioxide, red and white blood cell counts, hematocrit, hemoglobin, differential white blood cell count and platelet count) are monitored before and immediately after surgery, and every other day thereafter. Once normal bowel function commences, intravenous fluid therapy is terminated and animals are transferred to paddocks (i.e. 5-7 days post-surgery). Animals are maintained in individual pens (within sight of other animals), thereby reducing stress and the chance of sustaining damage to their catheter pouches (e.g. chewing by a penmate).
To inject treatments into individual loops, 5 ml of the treatment is injected into the catheter using a sterile syringe fitted with an 18 gauge, blunt-ended needle. To ensure the deposition of all of the treatment into the loop, the catheter is then injected with 3 ml of PBS.

### Critical points:

Procedures of crucial importance to the success of this surgical procedure include: (1) verifying that the intestine is not twisted on itself before establishing the anastomosis; (2) ensuring the integrity and patency of the anastomosis; (3) adequately securing the catheter to the loop wall with the ligature delineating the cranial end of the loop, but ensuring that the attachment does not affect the patency of the catheter; (4) minimizing intestinal handling and ensuring that exteriorized intestine is kept moist; (5) ensuring the appropriate catheter tension; (6) ensuring that the integrity of the catheter is not affected during muscle and skin suturing; (7) preventing gorging by sheep post-surgery (i.e. during the period of post-operative ileus) which can cause ruminal impaction and impairment of rumen function; and (8) ensuring that animals receive sufficient intravenous fluid therapy for the first 3-5 days post-surgery, that blood glucose concentrations are carefully monitored, and glucose concentrations in the intravenous fluids are adjusted if necessary to prevent hypoglycemia.
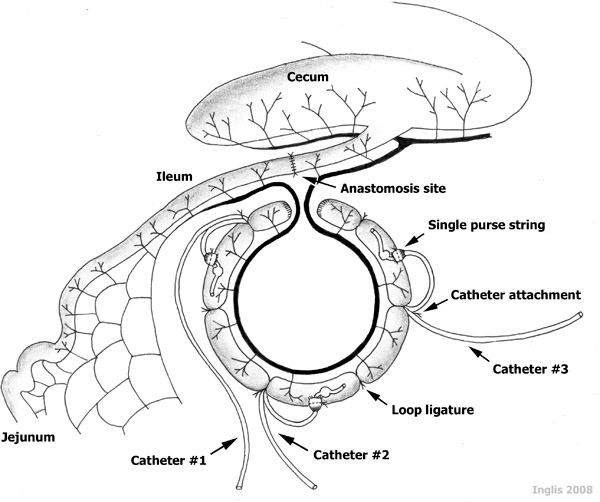
**Figure 1.** A schematic representation of catheterized ileal loops. Note the three 15 cm-long catheterized 'loops', the two 15 cm-long non-catheterized 'interspaces' established between the loops, and the two 5 cm-long terminal segments, all established within an 85 cm-long 'intestinal segment' of a sheep ileum.

## Disclosures

<p>All procedures on experimental animals were approved by the Institutional Animal Care Committee (Animal Use Protocol #0609) and followed the guidelines set by the Canadian Council of Animal Care.</p> <p>We have nothing to disclose.</p>

## Discussion

In this procedure, we were able to successfully establish catheters in intestinal loops established within the ileum of sheep. By adhering to stringent post-operative care, animals rapidly recovered from the procedure and resumed normal activity 5-7 days after surgery. We determined that the establishment of catheters within loops does not affect intestinal mucosal or immune function (data not shown) and animals can live for at least 8 wk after surgery. This procedure allows us to introduce multiple treatments into distinct immunological compartments over an extended interval, once animals have recovered from surgery, and to empirically measure the physiological and immunological effects of these treatments.

An important consideration for future experiments is the location of the intestinal segment within the small intestine. In this procedure we surgically created the intestinal segment within the ‘immunologically functional ileum’ as often defined by ileal (continuous) Peyer’s patches (5,6). This area can extend cranially up to 2 m beyond ileocecal fold. Although this region is considered anatomically as the jejunum, in the context of immunological function, it is ileum, highlighting the importance of considering function when describing intestinal regions.
